# Characteristics Associated with Pre-Exposure Prophylaxis Discussion and Use Among Transgender Women Without HIV Infection — National HIV Behavioral Surveillance Among Transgender Women, Seven Urban Areas, United States, 2019–2020

**DOI:** 10.15585/mmwr.su7301a2

**Published:** 2024-01-25

**Authors:** Elana Morris, Anna Teplinskaya, Evelyn Olansky, Jeffrey Kemp Rinderle, Johanna Chapin-Bardales, National HIV Behavioral Surveillance Among Transgender Women Study Group, National HIV Behavioral Surveillance Among Transgender Women Study Group, Narquis Barak, Kathleen A. Brady, Sarah Braunstein, Jasmine Davis, Sara Glick, Andrea Harrington, Jasmine Lopez, Yingbo Ma, Aleks Martin, Genetha Mustaafaa, Tanner Nassau, Gia Olaes, Jennifer Reuer, Alexis Rivera, William T. Robinson, Ekow Kwa Sey, Sofia Sicro, Brittany Taylor, Dillon Trujillo, Erin Wilson, Pascale Wortley

**Affiliations:** ^1^Behavioral and Clinical Surveillance Branch, Division of HIV Prevention, National Center for HIV, Viral Hepatitis, STD, and TB Prevention, CDC, Atlanta, Georgia; ^2^Social & Scientific Systems, Inc., Silver Spring, Maryland; ^3^Prevention Communication Branch, Division of HIV Prevention, National Center for HIV, Viral Hepatitis, STD, and TB Prevention, CDC, Atlanta, Georgia; CrescentCare; Philadelphia Department of Public Health; New York City Department of Health and Mental Hygiene; CrescentCare; University of Washington, School of Medicine, Division of Allergy and Infectious Diseases, Public Health – Seattle & King County, HIV/STD Program; Philadelphia Department of Public Health; New York City Department of Health and Mental Hygiene; Los Angeles County Department of Public Health; Public Health – Seattle & King County, HIV/STD Program; Georgia Department of Public Health; Philadelphia Department of Public Health; Los Angeles County Department of Public Health; Washington State Department of Health; New York City Department of Health and Mental Hygiene; Louisiana State University Health Science Center in New Orleans – School of Public Health, Louisiana Office of Public Health STD/HIV/Hepatitis Program; Los Angeles County Department of Public Health; San Francisco Department of Public Health; Georgia Department of Public Health; San Francisco Department of Public Health; San Francisco Department of Public Health; Georgia Department of Public Health

## Abstract

CDC recommends pre-exposure prophylaxis (PrEP) for transgender women who have sex with men and who report sexual behaviors that place them at substantial ongoing risk for HIV exposure, including those who engage in nonsterile syringe sharing. Providing transgender women with access to PrEP is a critical strategy for reducing HIV acquisition and ending the HIV epidemic. Survey results from the National HIV Behavioral Surveillance Among Transgender Women were used to assess characteristics associated with past-year discussions of PrEP with a health care provider and PrEP use. Bivariate analyses were conducted to assess the association between covariates (sociodemographic, HIV-associated characteristics, and gender-affirming care) and each outcome, accounting for sampling design. All covariates that were statistically significant at p<0.05 in the bivariate analyses were included in multivariate models, and manual backward elimination was used to obtain final models that retained statistically significant covariates. Among 902 transgender women from seven urban areas in the United States without HIV infection in the analyses, 57% had recently discussed PrEP with a health care provider, and 32% recently had used PrEP. In the final multivariate model, the following subgroups of transgender women were more likely to report recent PrEP use: those who identified as Black or African American or Hispanic or Latina, had two or more sex partners in the past 12 months, had condomless sex in the past 12 months, reported their last sex partner was infected with HIV, had condomless sex with their last sex partner whose HIV status was positive or unknown, ever had transgender-specific health care, and currently had transgender-specific health insurance coverage. Participants who were less likely to have recently used PrEP were those who wanted to but were not currently taking hormones and those aged <40 years. Providing increased access to gender-affirming care and training health care providers who serve transgender women to incorporate PrEP into existing services might increase PrEP use among transgender women.

## Introduction

Pre-exposure prophylaxis (PrEP) is highly effective at preventing HIV transmission when taken as prescribed (*1*) and is a critical strategy for the Ending the HIV Epidemic in the U.S. initiative (*2*). CDC recommends PrEP for transgender women who have sex with men and those who report sexual behaviors that place them at substantial ongoing risk for HIV exposure, including those who engage in nonsterile syringe sharing (*1*,*3*,*4*). Although many transgender women could benefit from PrEP, previous studies have identified barriers to PrEP use (e.g., poverty, lack of health insurance, homelessness, stigma, and discrimination), including discrimination within the health care system (*5*–*8*). Black or African American (Black) and Hispanic or Latina (Hispanic) transgender women who are among the groups most affected by HIV (*9*) also experience greater socioeconomic and structural barriers to PrEP use (*10*). (Persons of Hispanic origin might be of any race but are categorized as Hispanic; all racial groups are non-Hispanic.) In addition, qualitative studies of transgender women indicate that they might prioritize gender-affirming care over other health care because of its importance to their overall well-being (*6*,*11*). Previous studies have identified integrating gender-affirming care into HIV services as a strategy for increasing use of HIV services, including PrEP (*11*,*12*).

This report examines whether transgender women who report behaviors associated with HIV acquisition were more likely to have had discussions with their health care provider about PrEP and used PrEP. In addition, this report explores whether transgender women who had access to and used gender-affirming health care were more likely to have had discussions with their health care provider about PrEP and used PrEP. The findings in this report can help public health practitioners identify opportunities for increasing PrEP use among transgender women who might benefit from it.

## Methods

### Data Source

This report includes survey data from National HIV Behavioral Surveillance Among Transgender Women (NHBS-Trans) conducted by CDC during June 2019–February 2020 to assess behavioral risks, prevention usage, and HIV prevalence. Eligible participants completed an interviewer-administered questionnaire and were offered HIV testing. Definitions of demographics and social determinants of health are available in the overview and methodology report of this supplement (*13*). The NHBS-Trans protocol questionnaire and documentation are available at https://www.cdc.gov/hiv/statistics/systems/nhbs/methods-questionnaires.html#trans.

Applicable local institutional review boards in each participating project area approved NHBS-Trans activities. The final NHBS-Trans sample included 1,608 transgender women in seven urban areas in the United States (Atlanta, Georgia; Los Angeles, California; New Orleans, Louisiana; New York, New York; Philadelphia, Pennsylvania; San Francisco, California; and Seattle, Washington) using respondent-driven sampling. This analysis is limited to 902 participants with negative National HIV Behavioral Surveillance HIV test results. This activity was reviewed by CDC, deemed not research, and was conducted consistent with applicable federal law and CDC policy.*

### Measures

Outcome measures were PrEP discussion with a health care provider during the past 12 months and PrEP use during the past 12 months. Participants who reported not being aware of PrEP were considered to not have discussed PrEP or used PrEP during the past 12 months (Table 1). Definitions of certain covariates are available in the overview and methodology report of this supplement (*13*) and included sociodemographic, HIV-associated, and gender-affirming care characteristics. Sociodemographic characteristics assessed included age group (18–29, 30–39, and >40 years), race and ethnicity (American Indian or Alaska Native, Asian, Black, Hispanic, Native Hawaiian or other Pacific Islander, White, or persons of multiple races [persons of Hispanic origin might be of any race but are categorized as Hispanic; all racial groups are non-Hispanic]), education (less than high school, high school diploma or equivalent, some college or technical degree, and college degree or more), 2019 poverty level (at or below the Federal poverty level and above the Federal poverty level), and current health insurance.

**TABLE 1:** Variables, questions, measures, and analytic coding for HIV-associated gender-affirming care and pre-exposure prophylaxis characteristics among transgender women without HIV infection — National HIV Behavioral Surveillance Among Transgender Women, seven urban areas,* United States, 2019–2020

Variable	Question	Measure	Analytic coding
**HIV-associated characteristic**			
**Sexual behavior**			
Sex past 12 months	In the past 12 months, have you had oral, vaginal, or anal sex?	Number of sex partners past 12 months	0–1, 2–4, 5–9, or >10
Number of sex partners past 12 months	In the past 12 months, how many oral, vaginal, or anal sex partners have you had?		
Sex past 12 months	In the past 12 months, have you had oral, vaginal, or anal sex?	Anal or vaginal sex without a condom past 12 months	Yes or no
Vaginal sex past 12 months	In the past 12 months, have you had vaginal sex? By vaginal sex, I mean penis in the vagina, neovagina, or front hole.		
Insertive vaginal sex past 12 months	In the past 12 months, have you had insertive vaginal sex? By insertive vaginal sex, I mean where you put your penis in your partner’s vagina, neovagina, or front hole.		
Condomless insertive vaginal sex past 12 months	In the past 12 months, have you had insertive vaginal sex without a condom?		
Receptive vaginal sex past 12 months	In the past 12 months, have you had receptive vaginal sex? By receptive vaginal sex, I mean where your partner put their penis in your vagina, neovagina, or front hole.		
Condomless receptive vaginal sex past 12 months	In the past 12 months, have you had receptive vaginal sex without a condom?		
Anal sex past 12 months	In the past 12 months, have you had anal sex? By anal sex, I mean penis in the butt or back hole.		
Insertive anal sex past 12 months	In the past 12 months, have you had insertive anal sex? By insertive anal sex, I mean where you put your penis in your partner’s butt or back hole.		
Condomless insertive anal sex past 12 months	In the past 12 months, have you had insertive anal sex without a condom?		
Receptive anal sex past 12 months	In the past 12 months, have you had receptive anal sex? By receptive anal sex, I mean where your partner put their penis in your butt or back hole.		
Condomless receptive anal sex past 12 months	In the past 12 months, have you had receptive anal sex without a condom?		
Know last partner's HIV status	The last time you had sex, did you know your last partner’s HIV status?	Last partner's HIV status	HIV negative, HIV positive, or unknown
HIV status of last partner	What was your last partner’s HIV status?		
Vaginal sex with last partner	In the past 12 months, did you have vaginal sex with your last partner? By vaginal sex, I mean penis in the vagina, neovagina, or front hole.	Condomless anal or vaginal sex with last partner whose HIV status was positive or unknown	Yes or no
Condomless vaginal sex with last partner	In the past 12 months, did you have vaginal sex with your last partner without a condom?		
Anal sex with last partner	In the past 12 months, did you have anal sex with your last partner? By anal sex, I mean penis in the butt or back hole.		
Condomless anal sex with last partner	In the past 12 months, did you have anal sex with your last partner without a condom?		
Know last partner's HIV status	The last time you had sex, did you know your last partner’s HIV status?		
HIV status of last partner	What was your last partner’s HIV status?		
**Injection behavior**			
Ever injected drugs	Have you ever in your life shot up or injected any drugs other than those prescribed for you? By shooting up, I mean anytime you might have used a needle to inject drugs in your veins, under the skin, or in the muscle.	Injected drugs past 12 months	Yes or no
When last injected drugs	Now, think about the last time you injected any drug. When was that — how many days or months or years ago did you last inject?		
Ever taken hormones	Have you ever taken hormones for gender transition or affirmation?	Always used sterile needle to inject for gender affirmation past 12 months	Yes or no
Currently taking hormones	Are you currently taking hormones for gender transition or affirmation?		
Types of hormones used past 12 months	In the past 12 months, what forms of hormones did you take? You can choose more than one answer.		
Frequency of use of sterile needle to inject hormones past 12 months	In the past 12 months, when you had a hormone shot, how often was a new, sterile needle used? By a new, sterile needle, I mean a needle never used before by anyone, even you.		
Ever injected other gender affirming substances (e.g., injecting silicone)	Have you ever injected substances other than hormones to change your body to match your gender identity?		
Injection of other gender affirming substances past 12 months	In the past 12 months, have you injected these substances?		
Frequency of use of sterile needle to inject other gender affirming substances past 12 months	In the past 12 months, when you had these other injections, how often was a new, sterile needle used? By a new, sterile needle, I mean a needle never used before by anyone, even you.		
Ever injected drugs	Have you ever in your life shot up or injected any drugs other than those prescribed for you? By shooting up, I mean anytime you might have used a needle to inject drugs in your veins, under the skin, or in the muscle.	Nonsterile syringe sharing in the past 12 months	Yes or no
When last injected drugs	Now, think about the last time you injected any drug. When was that — how many days or months or years ago did you last inject?		
Frequency of use of a sterile needle to inject drugs past 12 months	In the past 12 months when you injected, how often did you use a new, sterile needle? By a new, sterile needle, I mean a needle never used before by anyone, even you.		
Frequency of use of previously used needle to inject drugs past 12 months	In the past 12 months, how often did you use needles that someone else had already injected with?		
Ever taken hormones	Have you ever taken hormones for gender transition or affirmation?		
Currently taking hormones	Are you currently taking hormones for gender transition or affirmation?		
Types of hormones used past 12 months	In the past 12 months, what forms of hormones did you take? You can choose more than one answer.		
Frequency of use of sterile needle to inject hormones past 12 months	In the past 12 months, when you had a hormone shot, how often was a new, sterile needle used? By a new, sterile needle, I mean a needle never used before by anyone, even you.		
Ever injected other gender affirming substances (e.g., injecting silicone)	Have you ever injected substances other than hormones to change your body to match your gender identity?		
Injection of other gender affirming substances past 12 months	In the past 12 months, have you injected these substances?		
Frequency of use of sterile needle to inject other gender affirming substances past 12 months	In the past 12 months, when you had these other injections, how often was a new, sterile needle used? By a new, sterile needle, I mean a needle never used before by anyone, even you.		
**Gender-affirming care characteristic**			
Ever comfortable with provider	Have you ever had a health care provider with whom you felt comfortable discussing gender-related issues?	Transgender-specific health care	Yes or no
Ever taken hormones	Have you ever taken hormones for gender transition or affirmation?	Hormone use for gender affirmation	Yes or no
Currently taking hormones	Are you currently taking hormones for gender transition or affirmation?		
Want to take hormones	Would you like to take hormones for gender transition or affirmation?		
**PrEP characteristic**			
PrEP awareness	Pre-exposure prophylaxis, or PrEP, is an antiretroviral medicine, such as Truvada, taken for months or years by a person who is HIV-negative to reduce the risk of getting HIV. Before today, have you ever heard of PrEP?	PrEP awareness	Yes or no
PrEP awareness	Pre-exposure prophylaxis, or PrEP, is an antiretroviral medicine, such as Truvada, taken for months or years by a person who is HIV-negative to reduce the risk of getting HIV. Before today, have you ever heard of PrEP?	PrEP discussion with health care provider past 12 months	Yes or no
Visited health care provider past 12 months	In the past 12 months, have you seen a doctor, nurse, or other health care provider?		
Discussed PrEP with a health care provider past 12 months	In the past 12 months, have you had a discussion with a health care provider about taking PrEP?		
PrEP awareness	Pre-exposure prophylaxis, or PrEP, is an antiretroviral medicine, such as Truvada, taken for months or years by a person who is HIV-negative to reduce the risk of getting HIV. Before today, have you ever heard of PrEP?	PrEP use past 12 months	Yes or no
PrEP use past 12 months	In the past 12 months, have you taken PrEP to reduce the risk of getting HIV?		

**Abbreviation:** PrEP = pre-exposure prophylaxis.

* Atlanta, GA; Los Angeles, CA; New Orleans, LA; New York City, NY; Philadelphia, PA; San Francisco, CA; and Seattle, WA.

Multiple sexual and injection HIV-associated characteristics also were assessed. Sexual behaviors included the number of sex partners during the past 12 months, anal or vaginal sex without a condom (hereafter referred to as condomless sex) during the past 12 months, last partner’s HIV status, condomless sex with last partner whose HIV status was positive or unknown, and received money or drugs in exchange for sex (hereafter referred to as exchange sex) during the past 12 months. Injection-related behaviors assessed during the past 12 months included injected drugs, always used sterile needle to inject for gender affirmation, and nonsterile syringe sharing to inject drugs or for gender affirmation (hereafter referred to as nonsterile syringe sharing). Gender-affirming care characteristics assessed included feeling comfortable discussing gender-related concerns with a provider during the past 12 months (hereafter referred to as transgender-specific health care), transgender-specific health insurance coverage (coverage of hormones for gender transition or affirmation), and hormone use (currently taking, not currently taking but want to take, and not currently taking and do not want to take).

### Analysis

This analysis was conducted in four steps using SAS software (version 9.4; SAS Institute). First, descriptive analyses were conducted to characterize the prevalence of sociodemographic, HIV-associated, and gender-affirming care characteristics, and the main PrEP outcomes among transgender women without HIV infection. Second, log-linked Poisson regression models with generalized estimating equations were used to assess the bivariate relations between each of the covariates and each outcome variable. Respondent-driven sampling method and network effects were accounted for in the models by clustering on recruitment chain and adjusting for urban area and network size to obtain adjusted prevalence ratios and 95% CIs. Third, two multivariate analyses were conducted, one for each PrEP outcome. Initially, all covariates with p<0.05 in the bivariate analysis were included in the full multivariate model for the respective outcome. Finally, manual backward elimination was used to create final models that retained statistically significant covariates (p<0.05). The multivariate models also included adjustment for urban area and network size and accounted for clustering by recruitment chain.

## Results

During the past 12 months among 902 transgender women without HIV infection, 42.5% had five or more sex partners, 64.6% had condomless sex, and 34.1% received money or drugs in exchange for sex (Table 2). More than one-third (41.1%) reported that they did not know the HIV status of their last sex partner, 3.2% reported their last partner’s HIV status was positive, and 14.5% had condomless sex with the last partner whose HIV status was positive or unknown. During the past 12 months, 5.0% injected drugs, 2.3% did not always use a sterile needle to inject for gender affirmation, and 3.5% shared a syringe to inject drugs or for gender affirmation. More than seven in 10 (73.2%) reported receiving transgender-specific health care within the past 12 months, and 70.2% currently had transgender-specific health insurance coverage. Approximately two thirds (68.1%) were currently taking hormones, 23.6% wanted to but were not currently taking hormones, and 8.2% were not currently taking hormones and did not want to take them.

**TABLE 2:** Number and percentage of transgender women without HIV infection,* by selected characteristics — National HIV Behavioral Surveillance Among Transgender Women, seven urban areas,^†^ United States, 2019–2020

Characteristic	No. (%)
**Sociodemographic**	
Age at interview, yrs	
18–29	358 (39.7)
30–39	253 (28.1)
≥40	290 (32.2)
Race and ethnicity^§^	
American Indian or Alaska Native	6 (0.7)
Asian	24 (2.7)
Black or African American	209 (23.2)
Native Hawaiian or other Pacific Islander	35 (3.9)
White	146 (16.2)
Multiracial	74 (8.2)
Hispanic or Latina	407 (45.2)
Education	
<High school	180 (20.0)
High school diploma or equivalent	317 (35.2)
Some college or technical degree	270 (30.0)
College degree or more	133 (14.8)
2019 poverty level^¶^	
At or below Federal poverty level	548 (61.4)
Above Federal poverty level	344 (38.6)
Current health insurance	
Yes	698 (77.4)
No	204 (22.6)
**HIV associated**	
**Sexual behavior**	
No. of sex partners past 12 months	
0–1	274 (30.8)
2–4	238 (26.7)
5–9	140 (15.7)
≥10	239 (26.8)
Anal or vaginal sex without a condom past 12 months	
Yes	581 (64.6)
No	319 (35.4)
Exchange sex past 12 months	
Yes	308 (34.1)
No	594 (65.9)
Last partner’s HIV status	
Negative	432 (55.7)
Positive	25 (3.2)
Unknown**	319 (41.1)
Condomless anal or vaginal sex with last partner whose HIV status was positive or unknown**	
Yes	112 (14.5)
No	661 (85.5)
**Injection behavior**	
Injected drugs past 12 months	
Yes	45 (5.0)
No	856 (95.0)
Always used sterile needle to inject for gender affirmation past 12 months	
Did not inject	494 (54.8)
Did inject, always used sterile needles	386 (42.8)
Did inject, did not always use sterile needles	21 (2.3)
Nonsterile syringe sharing past 12 months	
Yes	32 (3.5)
No	870 (96.5)
**Gender-affirming care**	
Transgender-specific health care^††^	
Yes	657 (73.2)
No	240 (26.8)
Transgender-specific health insurance coverage^§§^	
Yes	568 (70.2)
No	241 (29.8)
Hormone use for gender affirmation	
Currently taking hormones	604 (68.1)
Want to take hormones^¶¶^	209 (23.6)
Do not want to take hormones^¶¶^	74 (8.3)
**PrEP**	
Awareness	
Yes	827 (91.8)
No	74 (8.2)
Discussion with health care provider past 12 months	
Yes	510 (56.6)
No	391 (43.4)
Use past 12 months	
Yes	288 (32.0)
No	613 (68.0)
**Total**	**902 (100.0)**

**Abbreviation:** PrEP = pre-exposure prophylaxis.

* N = 902 participants with a negative National HIV Behavioral Surveillance HIV test result. Numbers might not sum to totals because of missing data.

^†^ Atlanta, GA; Los Angeles, CA; New Orleans, LA; New York City, NY; Philadelphia, PA; San Francisco, CA; and Seattle, WA.

^§^ Persons of Hispanic or Latina (Hispanic) origin might be of any race but are categorized as Hispanic; all racial groups are non-Hispanic.

^¶^ 2019 Federal poverty level thresholds were calculated on the basis of U.S. Department of Health and Human Services Federal poverty level guidelines (https://aspe.hhs.gov/topics/poverty-economic-mobility/poverty-guidelines/prior-hhs-poverty-guidelines-federal-register-references/2019-poverty-guidelines).

** Unknown includes participants who said they didn't know the HIV status of their last partner and two participants who reported that their last partner's HIV test result was indeterminate.

^††^ Transgender-specific health care was measured as “Ever having a provider with whom you are comfortable discussing gender related issues.”

^§§^ Transgender-specific health insurance coverage was measured as “Does your current health insurance cover hormones for gender transition or affirmation?”

^¶¶^ Participants who were not currently taking hormones were asked whether they want to take hormones for gender affirmation.

During the past 12 months, a majority of the 902 transgender women without HIV infection reported PrEP awareness (91.8%), more than half (56.6%) had discussed PrEP with a health care provider, and approximately one-third (32.0%) had used PrEP (Table 2). PrEP use was reported by 37.0% of those who had two or more sex partners, 38.0% of those who had condomless sex, 37.0% of those whose last sex partner’s HIV status was positive or unknown, 40.0% of those who received money or drugs in exchange for sex, and 44.0% of those who had condomless sex with last partner whose HIV status was positive or unknown (Figure). PrEP use also was reported by 33.0% of transgender women who injected drugs and by 44.0% who shared a syringe to inject drugs or for gender affirmation.

**FIGURE:**
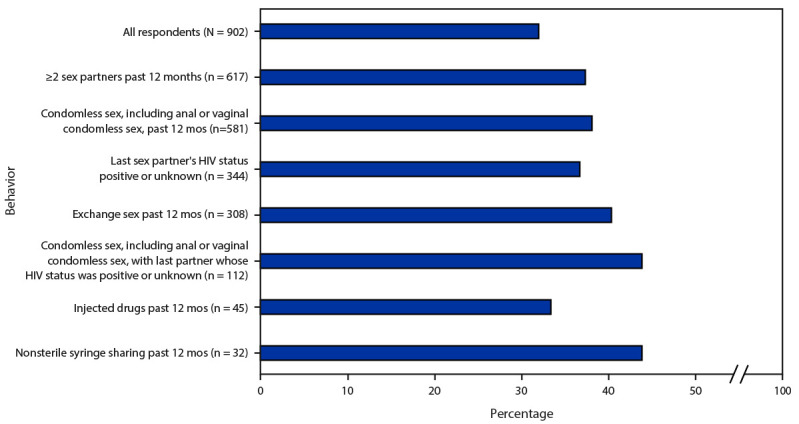
Percentage of transgender women without HIV infection who used pre-exposure prophylaxis during the past 12 months,* by sexual and injection behaviors^†^ — National HIV Behavioral Surveillance Among Transgender Women, seven urban areas,^§^ United States, 2019–2020^¶^ * N = 902 participants with a negative National HIV Behavioral Surveillance HIV testing result. ^†^ Behaviors are not mutually exclusive. ^§^ Atlanta, GA; Los Angeles, CA; New Orleans, LA; New York City, NY; Philadelphia, PA; San Francisco, CA; and Seattle, WA. ^¶^ Unknown includes participants who said they did not know the HIV status of their last partner and two participants who reported that their last partner’s HIV test result was indeterminate.

In the final multivariate model, transgender women who had condomless sex, who reported their last sex partner had HIV infection, or who received money or drugs in exchange for sex were more likely to have recently discussed PrEP than transgender women who did not report these behaviors (Table 3). Participants who had ever had transgender-specific health care and who currently had transgender-specific health insurance coverage were more likely to have recently discussed PrEP than participants lacking transgender-specific health care or insurance. Participants who wanted to but were not currently taking hormones were less likely to have recently discussed PrEP than participants currently taking hormones. Transgender women aged ≥40 years were less likely to have recently discussed PrEP than transgender women aged 18–29 years.

**TABLE 3:** Number, percentage, and adjusted prevalence ratios among transgender women without HIV infection discussing pre-exposure prophylaxis with a health care provider,* by selected characteristics — National HIV Behavioral Surveillance Among Transgender Women, seven urban areas,^†^ United States, 2019–2020

Characteristic	PrEP discussion with health care provider past 12 months				
	No. (%)	Bivariate association		Multivariate model	
		aPR^§^ (95% CI)	p value	aPR^§^ (95% CI)	p value
**Age at interview, yrs**					
18–29	221 (61.7)	Ref		Ref	
30–39	138 (54.5)	0.89 (0.78–1.02)	0.09	0.92 (0.81–1.04)	0.18
≥40	150 (51.7)	0.78 (0.69–0.87)	<0.01	0.85 (0.77–0.95)	<0.01
**Race and ethnicity^¶^**					
Black or African American	126 (60.3)	1.24 (0.92–1.67)	0.16	―**	―
White	71 (48.6)	Ref		―	―
Another race^††^	74 (53.2)	1.20 (0.86–1.68)	0.29	―	―
Hispanic or Latina	239 (58.7)	1.22 (0.93–1.61)	0.16	―	―
**Education**					
<High school	96 (53.3)	0.97 (0.74–1.26)	0.80	―	―
High school diploma or equivalent	189 (59.6)	1.11 (0.94–1.32)	0.21	―	―
Some college or technical degree	154 (57.0)	1.07 (0.91–1.26)	0.40	―	―
College degree or more	70 (52.6)	Ref		―	―
**Health insurance**					
Yes	413 (59.2)	1.24 (1.06–1.46)	<0.01	―	―
No	97 (47.5)	Ref		―	―
**2019 poverty level^§§^**					
At or below the Federal poverty level	321 (58.6)	1.02 (0.91–1.15)	0.70	―	―
Above the Federal poverty level	187 (54.4)	Ref		―	―
**Number of sex partners past 12 months**					
0–1	118 (43.1)	Ref		―	―
2–4	142 (59.7)	1.38 (1.18–1.60)	<0.01	―	―
5–9	87 (62.1)	1.41 (1.20–1.67)	<0.01	―	―
>10	155 (64.9)	1.51 (1.27–1.80)	<0.01	―	―
**Condomless anal or vaginal sex past 12 months**					
Yes	367 (63.2)	1.45 (1.20–1.74)	<0.01	1.18 (1.01–1.39)	0.04
No	142 (44.5)	Ref		Ref	
**Last partner’s HIV status**					
Negative	260 (60.2)	Ref		Ref	
Positive	20 (80.0)	1.27 (1.06–1.52)	0.01	1.21 (1.03–1.42)	0.02
Unknown^¶¶^	187 (58.6)	0.99 (0.90–1.08)	0.76	1.00 (0.91–1.09)	0.97
**Condomless anal or vaginal sex with last partner whose HIV status was positive or unknown** ^¶^					
Yes	66 (58.9)	0.94 (0.83–1.07)	0.36	―	―
No	399 (60.4)	Ref		―	―
**Exchange sex past 12 months**					
Yes	198 (64.3)	1.21 (1.09–1.35)	<0.01	1.13 (1.03–1.25)	0.01
No	312 (52.5)	Ref		Ref	
**Injected drugs past 12 months**					
Yes	28 (62.2)	1.10 (0.88–1.38)	0.40	―	―
No	482 (56.3)	Ref		―	―
**Nonsterile syringe sharing, past 12 months**					
Yes	23 (71.9)	1.28 (0.99–1.67)	0.06	―	―
No	487 (56.0)	Ref		―	―
**Transgender-specific health care**					
Yes	405 (61.6)	1.38 (1.21–1.58)	<0.01	1.25 (1.08–1.44)	<0.01
No	105 (43.8)	Ref		Ref	
**Transgender-specific health insurance coverage**					
Yes	361 (63.6)	1.33 (1.16–1.53)	<0.01	1.19 (1.05–1.35)	<0.01
No	114 (47.3)	Ref		Ref	
**Hormone use for gender affirmation**					
Currently taking hormones	386 (63.9)	Ref		Ref	
Want to take hormones***	88 (42.1)	0.69 (0.60–0.79)	<0.01	0.85 (0.73–0.99)	0.04
Do not want to take hormones***	29 (39.2)	0.63 (0.51–0.77)	<0.01	0.67 (0.40–1.12)	0.13
**Total**	**510 (56.6)**	**NA**	**NA**	**NA**	**NA**

**Abbreviations:** aPR = adjusted prevalence ratio; NA = not applicable; PrEP = pre-exposure prophylaxis; Ref = referent group.

* N = 510 participants with negative National HIV Behavioral Surveillance HIV test results and who discussed PrEP with a health care provider. Numbers might not sum to totals because of missing data.

^†^ Atlanta, GA; Los Angeles, CA; New Orleans, LA; New York City, NY; Philadelphia, PA; San Francisco, CA; and Seattle, WA.

^§^ Adjusted prevalence ratios in the bivariate and multivariate models accounted for respondent-driven sampling method by clustering on recruitment chain and adjusting for urban area and network size. The multivariate model also adjusted for the other variables with results listed in the column.

^¶^ Persons of Hispanic or Latina (Hispanic) origin might be of any race but are categorized as Hispanic; all racial groups are non-Hispanic.

** Dashes indicate variables not included in the multivariable model because there was not a statistically significant association in the bivariate association.

^††^ Includes participants who identified as American Indian or Alaska Native, Asian, Native Hawaiian or other Pacific Islander, or persons of multiple races.

^§§^ 2019 Federal poverty level thresholds were calculated on the basis of U.S. Department of Health and Human Services Federal poverty level guidelines (https://aspe.hhs.gov/topics/poverty-economic-mobility/poverty-guidelines/prior-hhs-poverty-guidelines-federal-register-references/2019-poverty-guidelines).

^¶¶^ Unknown includes participants that said they didn't know the HIV status of their last partner and two participants who reported that their last partner's HIV test result was indeterminate.

*** Participants who were not currently taking hormones were asked whether they want to take hormones for gender affirmation.

In the final multivariate model, transgender women who were Black or Hispanic were more likely to report recent PrEP use than White transgender women (Table 4). Participants who had two or more sex partners were more likely to report recent PrEP use compared with those with 0–1, 2–4, 5–9, and >10 partners. Transgender women who had condomless sex whose last sex partner had HIV infection and who had condomless sex with a last partner whose HIV status was positive or unknown were more likely to report recent PrEP use than transgender women who did not report these behaviors. Transgender women without HIV infection who had ever had transgender-specific health care and who currently had transgender-specific health insurance coverage were more likely to report recent PrEP use than transgender women lacking transgender-specific health care or insurance. Participants who wanted to but were not currently taking PrEP were less likely to recently use PrEP than participants who currently were taking hormones. Participants aged ≥40 years were less likely to have recently used PrEP than participants aged 18–29.

**TABLE 4:** Number, percentage, and adjusted prevalence ratios of transgender women without HIV infection who used pre-exposure prophylaxis,* by selected characteristics — National HIV Behavioral Surveillance Among Transgender Women, seven urban areas,^†^ United States, 2019–2020

Characteristic	PrEP use past 12 months				
	No. (%)	Bivariate association		Multivariate model	
		aPR^§^ (95% CI)	p value	aPR^§^ (95% CI)	p value
**Age at interview, yrs**					
18–29	123 (34.4)	Ref		Ref	
30–39	84 (33.2)	0.96 (0.78–1.17)	0.66	1.00 (0.81–1.23)	0.98
≥40	80 (27.6)	0.70 (0.57–0.86)	<0.01	0.79 (0.65–0.97)	0.02
**Race and ethnicity^¶^**					
Black or African American	64 (30.6)	1.33 (0.87–2.06)	0.19	1.42 (1.07–1.89)	0.02
White	33 (22.6)	Ref		Ref	
Another race**	41 (29.5)	1.47 (0.92–2.36)	0.11	1.38 (0.95–2.01)	0.09
Hispanic or Latina	150 (36.9)	1.72 (1.16–2.55)	<0.01	1.67 (1.26–2.21)	<0.01
**Education**					
<High school	51 (28.3)	1.00 (0.75–1.32)	0.98	—^††^	―
High school diploma or equivalent	110 (34.7)	1.28 (0.98–1.68)	0.07	―	―
Some college or technical degree	91 (33.7)	1.26 (0.86–1.84)	0.23	―	―
College degree or more	36 (27.1)	Ref		―	―
**Health insurance**					
Yes	240 (34.4)	1.35 (1.05–1.72)	0.02	―	―
No	48 (23.5)	Ref		―	―
**2019 poverty level^§§^**					
At or below Federal poverty level	178 (32.5)	0.96 (0.78–1.17)	0.67	―	―
Above Federal poverty level	109 (31.7)	Ref		―	―
**Number of sex partners past 12 months**					
0–1	53 (19.3)	Ref		Ref	
2–4	78 (32.8)	1.70 (1.27–2.27)	<0.01	1.35 (1.10–1.66)	<0.01
5–9	54 (38.6)	1.94 (1.40–2.69)	<0.01	1.60 (1.25–2.04)	<0.01
≥10	98 (41.0)	2.18 (1.54–3.10)	<0.01	1.48 (1.10–1.98)	<0.01
**Condomless anal or vaginal sex past 12 months**					
No	66 (20.7)	Ref		Ref	
Yes	221 (38.0)	1.88 (1.47–2.39)	<0.01	1.57 (1.28–1.93)	<0.01
**Last partner’s HIV status**					
Negative	140 (32.4)	Ref		Ref	
Positive	14 (56.0)	1.61 (1.19–2.18)	<0.01	1.49 (1.09–2.05)	0.01
Unknown^¶¶^	112 (35.1)	1.10 (0.84–1.45)	0.47	0.99 (0.75–1.31)	0.94
**Condomless anal or vaginal sex with last partner whose HIV status was positive or unknown**					
Yes	49 (43.8)	1.30 (1.02–1.66)	0.03	1.40 (1.06–1.83)	0.02
No	216 (32.7)	Ref		Ref	
**Exchange sex past 12 months**					
Yes	124 (40.3)	1.44 (1.15–1.79)	<0.01	―	―
No	164 (27.6)	Ref		―	―
**Injected drugs past 12 months**					
Yes	15 (33.3)	1.04 (0.71–1.54)	0.83	―	―
No	273 (31.9)	Ref		―	―
**Nonsterile syringe sharing past 12 months**					
Yes	14 (43.8)	1.43 (1.00–2.07)	0.053	―	―
No	274 (31.5)	Ref		―	―
**Transgender-specific health care**					
Yes	240 (36.5)	1.71 (1.39–2.10)	<0.01	1.42 (1.07–1.88)	0.02
No	48 (20.0)	Ref		Ref	
**Transgender-specific health insurance coverage**					
Yes	216 (38.0)	1.48 (1.20–1.83)	<0.01	1.39 (1.06–1.83)	0.02
No	56 (23.2)	Ref		Ref	
**Hormone use for gender affirmation**					
Currently taking hormones	230 (38.1)	Ref		Ref	
Want to take hormones***	41 (19.6)	0.57 (0.42–0.76)	<0.01	0.73 (0.57–0.94)	0.02
Do not want to take hormones***	12 (16.2)	0.46 (0.31–0.68)	<0.01	0.72 (0.37–1.41)	0.34
**Total**	**288 (32.0)**	**NA**	**NA**	**NA**	**NA**

**Abbreviations:** aPR = adjusted prevalence ratio; NA = not applicable; PrEP = pre-exposure prophylaxis.

* N = 288 participants with negative National HIV Behavioral Surveillance HIV test results who used PrEP. Numbers might not sum to totals because of missing data.

^†^ Atlanta, GA; Los Angeles, CA; New Orleans, LA; New York City, NY; Philadelphia, PA; San Francisco, CA; and Seattle, WA.

^§^ Adjusted prevalence ratios in the bivariate and multivariate models accounted for respondent-driven sampling method by clustering on recruitment chain and adjusting for urban area and network size. The multivariate model also adjusted for the other variables with results listed in the column.

^¶^ Persons of Hispanic or Latina (Hispanic) origin might be of any race but are categorized as Hispanic; all racial groups are non-Hispanic.

** Includes participants who identified as American Indian or Alaska Native, Asian, Native Hawaiian or other Pacific Islander, or persons of multiple races.

^††^ Dashes indicate variable not included in the multivariable model because there was not a statistically significant association in the bivariate association.

^§§^ 2019 Federal poverty level thresholds were calculated on the basis of U.S. Department of Health and Human Services Federal poverty level guidelines (https://aspe.hhs.gov/topics/poverty-economic-mobility/poverty-guidelines/prior-hhs-poverty-guidelines-federal-register-references/2019-poverty-guidelines).

^¶¶^ Unknown includes participants who said they didn’t know the HIV status of their last partner and two participants who reported that their last partner’s HIV test result was indeterminate.

*** Participants who were not currently taking hormones were asked whether they wanted to take hormones for gender affirmation.

## Discussion

The findings in this report are encouraging. Awareness of PrEP in this sample was high (92.0%) compared with previous studies (*6*,*14*–*16*), which might indicate progress in increasing awareness of PrEP as an HIV prevention option for transgender women. In addition, transgender women without HIV infection who engaged in sexual behaviors that might be associated with HIV acquisition were more likely to discuss PrEP with a health care provider and use it.

Further, Hispanic transgender women were 1.7 times more likely and Black transgender women were 1.4 times more likely than their White counterparts to use PrEP. These racial and ethnic differences in PrEP use are important because of the substantial racial and ethnic inequities in HIV infection among transgender women. Estimates of HIV prevalence among Black transgender women range from 44% to 62% and from 26% to 35% for Hispanic transgender women, compared with from 7% to 17% among White transgender women (*9*). In addition, a recent study estimated that approximately 88% of Black and Latina transgender women could benefit from PrEP, indicating a potential substantial need for PrEP among transgender women of color (*17*).

Exchange sex did not remain statistically significant in the final multivariate model for PrEP use, whereas other behaviors (e.g., number of sex partners and condomless sex) did stay in the final model. Exchange sex on its own is not a risk for HIV acquisition; therefore, it is reasonable that a behavior that is more directly associated with HIV acquisition (e.g., condomless sex) remained associated with PrEP use and exchange sex did not. Yet, one in three transgender women in the study reported exchange sex during the past year, and often sex workers experience social, legal, and economic marginalization that has been associated with HIV transmission (*5*,*18*) that can be mitigated (e.g., one systematic review found that providing sensitivity training to law enforcement officials resulted in sex workers reporting considerably greater ability to negotiate safer sex practices and less sexual or physical violence from clients) (*19*). However, in many cases, these challenges are difficult to mitigate, and PrEP could play an important role in supporting a person’s autonomy to prevent HIV acquisition.

Although prevalence of injection-related behaviors (i.e., injection drug use or nonsterile injection of gender-affirming procedures) was low in this sample, nonsterile injection can be a source of HIV acquisition for transgender women (*20*–*22*). Access to sterile injection equipment can be a problem for transgender women who do not have access to gender-affirming treatments (e.g., hormones or silicone) through the health care system and therefore might use nonprescribed treatments (*16*,*21*,*22*). Ensuring access to sterile syringes for transgender women who might inject drugs or use syringes for gender-affirming treatments is important. Syringe services programs (https://www.cdc.gov/ssp/index.html) are an important part of HIV prevention strategies (*23*) and might offer syringes that are specifically needed for gender-affirming injection in addition to existing sterile syringe services that lower HIV risk related to injection drug use (*24*,*25*). Further, CDC recommends assessing risk for nonsterile injection use and providing PrEP to persons who report nonsterile injection behaviors (*1*).

Despite a high prevalence of PrEP awareness and use in the survey participants, overall, only one third of transgender women used PrEP during the past 12 months. When looking independently at the percentage of transgender women who used PrEP by sexual and injection behaviors, PrEP use ranged from 33.0% to 44.0%, respectively, among participants reporting each of the behaviors. The findings from this analysis are similar to existing evidence that also indicates suboptimal PrEP use among transgender women who might benefit from its use (*6*,*11*,*26*–*29*). Concerns about interactions between hormones and PrEP, missed opportunities within the health care system, and mistrust of the health care system because of experiences of discrimination, have been reasons noted for low PrEP use among transgender women (*5*,*6*,*10*,*29*,*30*).

Transgender women who had access to and were using gender-affirming care, including transgender women taking hormones, were more likely to have discussions with a health care provider about PrEP and use PrEP; the strength of these associations remained in the multivariate model. Other studies have identified that providing gender-affirming care can increase engagement in HIV prevention services and improve viral suppression outcomes (*6*,*28*,*31*,*32*). Current health insurance, which typically is a measure of health care access, did not remain a statistically significant measure in the multivariate models. To what extent gender-affirming care initiates transgender patients into health care systems where HIV services are more accessible or whether increasing trust in gender-affirming health care systems fosters improved engagement in HIV prevention is unclear. Evidence from a qualitative study of transgender women indicates that both might be operating; having positive experiences within the health care system and combining PrEP and gender-related health care visits were both identified as facilitators for PrEP use (*33*). Another study found that, among transgender women with HIV infection, gender affirmation and health empowerment moderated the negative effects of discrimination to improve viral suppression (*32*). The specific dynamics by which gender-affirming care enhances engagement and retention could benefit from further study. Regardless of the underlying mechanisms, these findings indicate that access to and use of gender-affirming care might be an important way to reach transgender women to provide other prevention health services, including PrEP.

In addition to providing gender-affirming care, certain recent evidence also has pointed to the importance of providing support services (e.g., transportation and legal and mental health services to improve PrEP initiation, retention, and adherence for transgender women) (*34*). Moreover, interventions that include shared decision making that are community led and culturally appropriate also have been identified as promising approaches to increasing PrEP use among Black and Hispanic transgender women (*16*,*34*,*35*). CDC is pursuing a variety of prevention strategies including funding transgender-focused prevention programs, researching HIV interventions, building the capacity of the HIV workforce, and developing and disseminating social marketing campaigns. One example is a pilot program (https://www.cdc.gov/hiv/funding/announcements/ps22-2209/index.html) that funds community-based organizations to develop community-to-clinic health models to provide access to integrated status-neutral HIV prevention and care services, gender-affirming services that include access to or referral to hormone therapy, and primary health care. Another example is CDC’s evidence-based Let’s Stop HIV Together (https://www.cdc.gov/stophivtogether/index.html) social marketing campaign that includes tailored resources for transgender audiences. The campaign’s HIV Nexus (https://www.cdc.gov/hiv/clinicians/index.html) clinician portal (https://www.cdc.gov/endhiv/index.html) includes the Transforming Health website (https://www.cdc.gov/hiv/clinicians/transforming-health/index.html) where CDC offers educational materials for health care and social service providers to help them improve care for transgender persons with HIV infection and make clinical environments more welcoming to transgender patients.

## Limitations

General limitations for the NHBS-Trans are available in the overview and methodology report of this supplement (*13*). The findings in this report are subject to at least four additional limitations. First, the sample is not representative of transgender women living outside of the seven urban areas. The cities included in this analysis are urban areas that might have more resources for engaging transgender women in PrEP than in other geographic areas. Because of the hard-to-reach nature of transgender women, the data might not be representative of all transgender women residing in the seven urban areas. Second, the data are cross-sectional, and therefore directionality between covariates and PrEP discussions and use cannot be determined. Third, the sexual and injection behaviors included in the analysis are not in exact alignment with CDC clinical practice guidelines for PrEP (*1*); for example, the time frame for assessed behaviors differed (e.g., past 12 months and last sex) and the survey did not assess a recent diagnosis of sexually transmitted infection. Finally, the PrEP questions included in NHBS-Trans were limited to use within the past 12 months and did not include assessment of continued or effective use of PrEP, which are critical for the prevention of HIV.

## Conclusion

Half of transgender women without HIV infection had discussed PrEP with their health care provider during the past year, and one third had used PrEP. Although transgender women who reported sexual behaviors that are associated with HIV acquisition were more likely to have PrEP discussions with their health care provider and use PrEP, many who could benefit from PrEP were not using it. Because transgender women are one of the groups most affected by HIV (*9**,**36*), providing access to PrEP is critical to reducing HIV acquisition risk among transgender women and to reaching the goals for Ending the HIV Epidemic in the United States (*2*). Use of gender-affirming care was associated with having discussions about PrEP with a health care provider and PrEP use. Improving access to gender-affirming care for transgender women and training health care providers that serve transgender women to incorporate HIV prevention, including PrEP, into their services are strategies that might help increase PrEP use among transgender women.

## Footnotes

* 45 C.F.R. part 46, 21 C.F.R. part 56; 42 U.S.C. Sect. 241(d); 5 U.S.C. Sect. 552a; 44 U.S.C. Sect. 3501 et seq.
